# Supplementary material for: In Silico Design of a Multiepitope Vaccine Against Intestinal Pathogenic Escherichia coli Based on the 2011 German O104:H4 Outbreak Strain Using Reverse Vaccinology and an Immunoinformatic Approach
Source: Diseases. 2025 Aug 13;13(8):259. doi: 10.3390/diseases13080259 (PMC12385595; doi:10.3390/diseases13080259)
Supplement: Supplementary file 1 [file diseases-13-00259-s001.zip › diseases-3792883-Supplementary Materials .pdf]

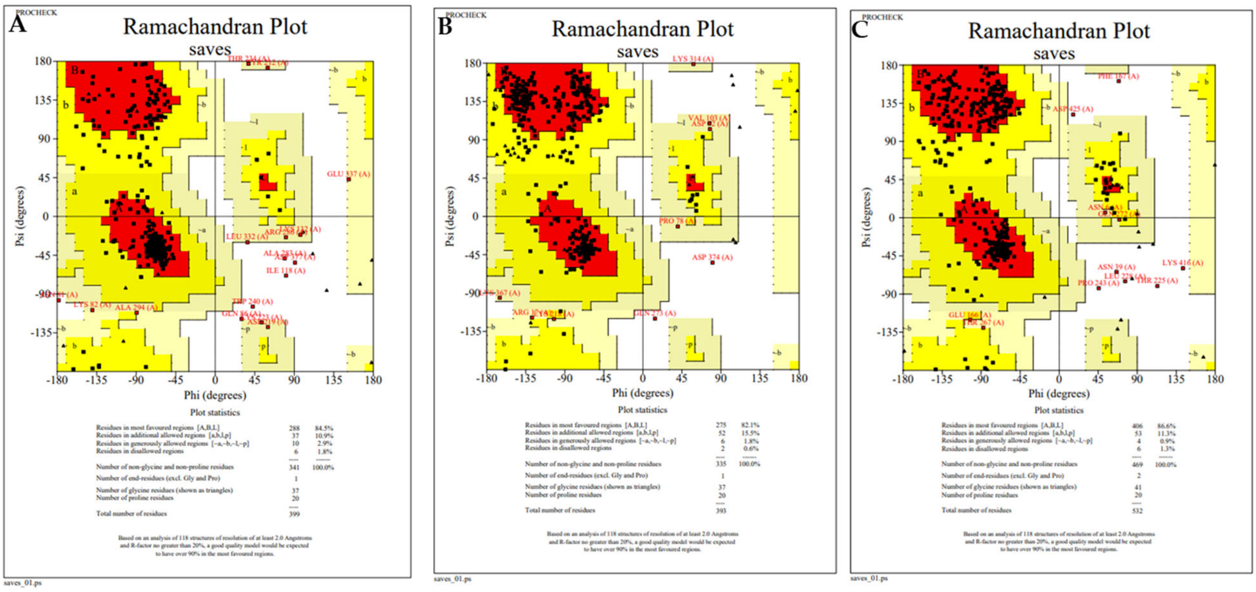

Figure S1. Ramachandran plots of the refined tertiary structures of **(A)** *Ecoepvc1*, **(B)** *Ecoepvc2*, and **(C)** *Ecoepvc4*.
